# Surgical Treatment of Osteosarcoma Induced Distant Pre‐Metastatic Niche in Lung to Facilitate the Colonization of Circulating Tumor Cells

**DOI:** 10.1002/advs.202207518

**Published:** 2023-08-16

**Authors:** Fan Tang, Yan Tie, Tian‐Xia Lan, Jing‐Yun Yang, Wei‐Qi Hong, Si‐Yuan Chen, Hou‐Hui Shi, Long‐Qing Li, Hao Zeng, Li Min, Yu‐Quan Wei, Chong‐Qi Tu, Xia‐Wei Wei

**Affiliations:** ^1^ Laboratory of Aging Research and Cancer Drug Target State Key Laboratory of Biotherapy National Clinical Research Center for Geriatrics West China Hospital Sichuan University No. 17, Block 3, Southern Renmin Road Chengdu Sichuan 610041 People's Republic of China; ^2^ Department of Orthopedics Orthopedic Research Institute West China Hospital Sichuan University No. 17, Block 3, Southern Renmin Road Chengdu Sichuan 610041 People's Republic of China

**Keywords:** circulating tumor cells, osteosarcoma, pre‐metastatic niche, pulmonary metastasis, surgical treatment

## Abstract

Recently, the major challenge in treating osteosarcoma patients is the metastatic disease, most commonly in the lungs. However, the underlying mechanism of recurrence and metastasis of osteosarcoma after surgical resection of primary tumor remains unclear. This study aims to investigate whether the pulmonary metastases characteristic of osteosarcoma is associated with surgical treatment and whether surgery contributes to the formation of pre‐metastatic niche in the distant lung tissue. In the current study, the authors observe the presence of circulating tumor cells in patients undergoing surgical resection of osteosarcoma which is correlated to tumor recurrence. The pulmonary infiltrations of neutrophils and Gr‐1^+^ myeloid cells are characterized to form a pre‐metastatic niche upon the exposure of circulating tumor cells after surgical resection. It is found that mitochondrial damage‐associated molecular patterns released from surgical resection contribute to the formation of pre‐metastatic niche in lung through IL‐1β secretion. This study reveals that surgical management for osteosarcoma, irrespective of the primary tumor, might promote the formation of postoperative pre‐metastatic niche in lung which is with important implications for developing rational therapies during peri‐operative period.

## Graphical abstract

In this study, the authors analyze the pulmonary microenvironment using a mouse model of osteosarcoma subjected to surgical trauma, examining its influence on circulating tumor cells (CTCs). They reveal the involvement of mitochondrial damage‐associated molecular patterns (DAMPs) in this process. Furthermore, the authors show that IL‐1β‐mediated anti‐inflammatory interventions can alleviate the formation of a pre‐metastatic niche in the lung induced by surgeries and reduce subsequent colonization of circulating tumor cells.

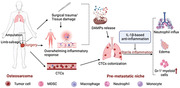

## 1 Introduction

Surgical resection is the cornerstone of treatment for malignant solid tumors. However, despite the curative intent of the procedure, the incidence of postoperative metastasis remains high, ranging from 20% to 60%.^[^ ^1^ ^]^ This issue is also observed in osteosarcoma, the most common primary malignant bone tumor. Neoadjuvant and adjuvant chemotherapies combined with surgery have improved overall survival to over 60% in patients with early‐stage disease.^[^ ^2^ ^]^ However, the current challenge in treating osteosarcoma is the high incidence of metastatic relapse, particularly in the lungs. It is reported that the first relapse occurs at a median interval of 1.7 years from the diagnosis, and with over 80% metastatic relapse cases involve metastasis to the lungs.^[^ ^3^ ^]^ Although the role of primary tumor cells in regulating the pulmonary microenvironment has been studied, the mechanisms underlying postoperative metastasis of osteosarcoma, especially relapse after primary tumor resection, remain unclear.^[^ ^4^ ^]^

Increasing evidence suggests that circulating tumor cells can predict prognosis and tumor burden, including in patients who have undergone tumor surgeries.^[^ ^5^ ^]^ Several studies monitored the circulating tumor cells in the murine osteosarcoma model for developing lung metastasis, however, it remains unclear whether circulating tumor cells are accidentally trapped by the lungs or selectively targeted to promote metastasis.^[^ ^6^ ^]^ Importantly, the settlement and colonization of circulating tumor cells in distant metastatic target sites critically depends on the formation of pre‐metastatic niches in the local microenvironment.^[^ ^7^ ^]^ One of the main features of pre‐metastatic niches is the inflammation characterized with infiltration of neutrophils and monocytes.^[^ ^8^ ^]^ For instance, inflammation in the lungs induced by lipopolysaccharide (LPS) could form a pre‐metastatic niche through the influx of bone marrow‐derived neutrophils and production of inflammatory mediators that enhance the growth of metastatic cells.^[^ ^9^ ^]^

Besides infection, tissue injury and cell damage can trigger inflammation as a key driver of the immune response.^[^ ^10^ ^]^ The surgical treatment of osteosarcoma was accompanied by extensive excision and soft‐tissue reconstructions, leading to severe tissue injuries, which might result in overwhelming inflammation and post‐operative complications.^[^ ^11^ ^]^ A previous study has observed that wound‐healing process after surgery could induce systemic inflammatory response favorable for tumor growth in a mouse model.^[^ ^12^ ^]^ However, whether the local surgical resection drives the distant pre‐metastatic niche formation for tissue specific metastatic relapse remains unclear, especially in the case of osteosarcoma. Given that lungs are highly susceptible to inflammation and the most commonly affected organ in osteosarcoma metastasis, this study aimed to elucidate the underlying mechanism of postoperative lung metastasis of osteosarcoma by exploring how surgical treatment resulted in a distant regulation in lung, rendering a tumor supportive inflammatory microenvironment for the circulating tumor cells. Moreover, the critical role of damage associated molecule patterns (Ds) released during and following surgical treatment in promoting the distant pre‐metastatic niche responsible for lung metastasis was also studied.

## 2 Results

### 2.1 Systemic Inflammation and Circulating Tumor Cells were Detected in Osteosarcoma Patients after Surgeries

**Figure 1:**
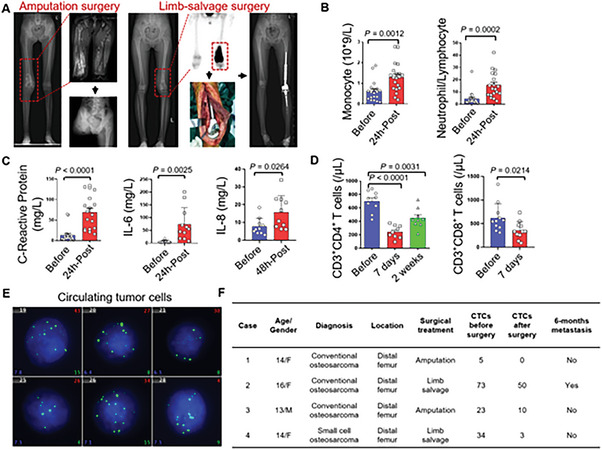
Systemic inflammatory response, impaired cellular immunity, and circulating tumor cells were detected after osteosarcoma surgeries. A) clinical images from a 13‐year‐old male with osteosarcoma in the distal femur, receiving amputation surgery, and a 14‐year‐old female in the distal femur, receiving limb‐salvage surgery. B) Statistics of circulating monocytes and neutrophil/lymphocytes ratio before and after osteosarcoma surgeries. Data are presented as means ± SEM (Student's *t*‐test; *n* = 19 to 20). C) Comparison of C‐reactive protein, pro‐inflammatory cytokine IL‐6, and IL‐8 before and after surgeries. Data are presented as means ± SEM (Student's *t*‐test; *n* = 5 to 11). D) Statistics of the CD3^+^CD4^+^ T cells and CD3^+^CD8^+^ T cells before and after osteosarcoma surgeries. Data are presented as means ± SEM (Student's *t*‐test; *n* = 9 to 11). E) fluorogram reflecting circulating tumor cells in case 1 before surgery (Green fluorogram representing tumor cells with mesenchymal origination). F) Clinical information of included patients with osteosarcoma for circulating tumor cells evaluation before and after surgeries.

The surgical treatments for primary osteosarcoma include amputation surgery and limb‐salvage surgery, both of which involve extensive excision and cause significant tissue injuries (**Figure** **1**A). The inflammation and impaired cellular immunity are associated with the survival and growth of tumor cells.^[^ ^13^ ^]^ In order to further analyze the systemic response to surgery, we initially assessed the inflammatory status in the postoperative period. We observed an increase in the number of monocytes and the neutrophil/lymphocyte ratio after osteosarcoma surgeries (Figure 1B). In addition, the C‐reactive protein, which would elevate the response to infection or injury, also significantly increased (Figure 1C). Among cytokines, the pro‐inflammatory cytokine IL‐6 immediately increased after surgeries, followed by the increase of chemo‐cytokine IL‐8 (Figure 1C). Disruption of the immune system caused by surgical treatment for solid tumors has been reported as a critical factor for early postoperative relapse.^[^ ^12^ ^]^ Thus, the alteration of cellular immunity before and after surgeries for patients with osteosarcoma is evaluated. In detail, CD3^+^CD4^+^ helper T cells, and CD3^+^CD8^+^ cytotoxic T cells (Figure 1D) decreased most prominently on the seventh day after surgery but recovered within two weeks. These changes in cellular immunity corresponded with the previous report that the first two weeks after surgeries were critical for therapeutic decision‐making.^[^ ^14^ ^]^

The extravasation of tumor cells from primary sites into the circulation system, known as circulating tumor cells, represents the initial step of distant metastasis.^[^ ^15^ ^]^ The perioperative level of circulating tumor cells is closely related to recurrence and metastasis after tumor resection.^[^ ^16^ ^]^ Thus, we designed seven PCR probes to detect the proposed tumor cells and clusters and calculated the number of circulating tumor cells before and after surgeries (Figure 1E). All of the included patients received biopsy and neoadjuvant chemotherapy before surgeries, and preoperative chest CT scans did not reveal any metastases. Four patients with Enneking Stage IIb disease exhibited the presence of circulating tumor cells before surgeries. Moreover, circulating tumor cells were still detectable in three patients one to three days after surgery, although the number of circulating tumor cells reduced (Figure 1F). It is proposed that circulating tumor cells were closely related to long‐term survival, with a small group of circulating tumor cells settling and growing to metastases at the distant organ, although most circulating tumor cells became latent or eliminated by the host.^[^ ^17^ ^]^

### 2.2 Surgical Treatment Elicited an Alteration of Immunological Transcriptome in Lung

**Figure 2:**
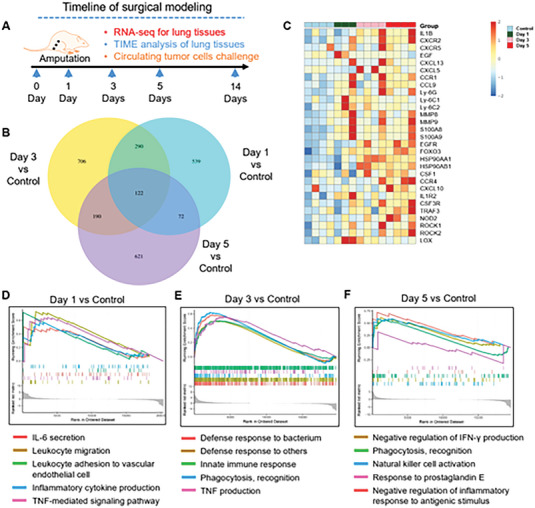
Surgery elicits an inflammation response with immune suppression in the lung. A) The experimental design on surgical trauma mice model. B) The Venn diagram showing the number of differentially expressed genes comparing to control group at different time points. C) The heatmap of the differential expressing genes of lungs after surgeries when compared with the control group. D) Signaling pathway enrichment analysis was performed using Gene Ontology (GO) analysis. Significantly enriched (nominal P < 0.05) pathways in lungs at Day 1 after surgeries are plotted by enrichment score. E) Significantly enriched (nominal P < 0.05) pathways in lungs at Day 3 after surgeries and control group are plotted by enrichment score. F) Significantly enriched (nominal P < 0.05) pathways in lungs at Day 5 after surgeries and control group are plotted by enrichment score.

The colonization of circulating tumor cells at distant organs relies on the presence of pre‐metastatic niches in host organs.^[^ ^18^ ^]^ To investigate the effects of surgical treatment on the lungs, we established a surgical mouse model that mimicked the clinical extent of surgical trauma as reported previously (**Figure** **2**A).^[^ ^19^ ^]^ Subsequently, we collected fresh lung tissues from traumatized mice during the operative period and performed RNA‐seq analysis. The transcriptome analysis revealed dynamic responses subject to surgical trauma, with distinct gene expression profiles (Figure 2B,C). On the first day (Day 1) after surgery, the lungs exhibited an inflammatory response pattern, with upregulated signals of IL‐6 secretion, TNF‐mediated pathways, as well as cytokine production and leukocyte migration involved in inflammatory response (Figure 2D). In addition, the leukocytes presented increased adhesion to vascular endothelial cells (Figure 2D). By Day 3 after surgeries, the lungs initiated the innate immune response, primarily targeting bacteria and other organisms, which we hypothesized that there might be released damage‐associated molecular patterns due to the surgical injury (Figure 2E). On Day 5 after surgeries, the lungs exhibited an immunosuppressive transcriptome with upregulated signals associated with the negative regulation of inflammatory response to antigenic stimulus and IFN‐γ secretion (Figure 2F). Also, at this time, the inflammation still existed in the lungs subject to surgical trauma, as evidenced by increased signals related to prostaglandin E (Figure 2F).

### 2.3 Surgical Treatment Induced Post‐operatively Transient Inflammation in Lung

**Figure 3:**
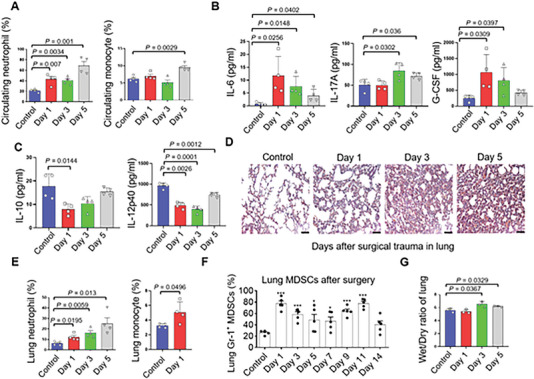
Gr‐1^+^ myeloid cells influx into the lung subject to surgical treatment. A) Statistics of the number of circulating neutrophils and monocyte before and after surgery in mice. Data are presented as means ± SEM (Student's *t*‐test; *n* = 4). B,C) Cytokines in serum using Luminex (n = 4). D) H&E staining of the lungs in mice before and after surgeries (Scale bars, 50 µm). E) Flowcytometry analysis of neutrophils and monocytes in lungs before and after surgeries (n = 4). F) Statistics of MDSCs in the lungs during the perioperative period (n = 5). G) The wet to dry ratio of the lung in mice before and after surgeries (n = 3). Graphs show mean ± SEM. *P < 0.05; **P < 0.01; ***P < 0.001. ns, not significant.

Then, we collected the fresh blood samples and lung tissues during the perioperative period to analyze the local pulmonary microenvironment. Similar to the response observed in patients undergoing surgery, circulating neutrophils and inflammatory monocytes also increased in mice after surgeries, respectively (**Figure** **3**A). Besides, pro‐inflammatory cytokines such as IL‐6, IL‐17A, and G‐CSF in circulation elevated after surgical trauma (Figure 3B). However, circulating anti‐inflammatory cytokines like IL‐10 and IL‐12p40 decreased subject to surgery (Figure 3C). Histological examination with corresponding H&E staining revealed inflammatory infiltration in the lungs, with the most prominent inflammation observed on Day 5 (Figure 3D). Furthermore, similar to the effects of LPS stimulation, neutrophils influx into the lungs after limb surgery, with the number of which reaching peak at Day 3 or Day 5 (Figure 3E). The inflammatory monocyte elevated in the lung at the early stage after surgery (Figure 3E). The M2‐macrophages in the lung did not elevate after surgeries (Figure S1A). Myeloid‐derived suppressor cells (MDSCs) are responsible for pre‐metastatic niches formation in distant organs after surgeries.^[^ ^20^ ^]^ Flow cytometry analysis of blood samples and lung tissues revealed the dynamics of MDSCs in response to surgeries. MDSCs traveled in circulation during the two‐week perioperative period in mice after surgeries (Figure S1B). In the lungs, MDSCs marked by CD11b^+^Gr‐1^+^ sustained for up to two weeks after surgeries (Figure 3F), producing immune suppression cytokine TGF‐β (Figure S1C). Besides, the pro‐inflammatory cytokine IL‐1β secreted by MDSCs also increased after surgery (Figure S1D). In addition, the wet to dry weight ratio representing the edema of the lung also increased on Day 3 and Day 5 after surgeries (Figure 3G). However, these inflammatory infiltrations diminished after two weeks, indicating a transient nature (Figure S1E).

### 2.4 Surgical Treatment Promoted Pulmonary Pre‐metastatic Niche Formation upon Circulating Tumor Cell Exposure

**Figure 4:**
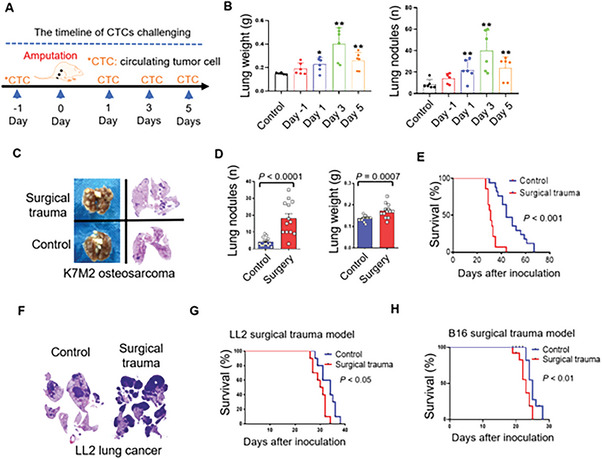
Lung becomes vulnerable to circulating tumor cells subject to surgical treatment. A) The experimental design of surgery on the colonization of circulating tumor cells in lung. B) Statistics of the pulmonary weight and the number of nodules in lung challenged with osteosarcoma cells (5 × 10^5^ cells per mouse) one day before surgery, one day after surgery, three days after surgeries, and five days after surgeries (n = 6). C) Images of the lung and the corresponding H&E staining of mice received surgery and without surgery. D) Statistics of the number and weight of pulmonary metastases in mice who received surgery and without surgery (n = 13) (5 × 10^5^ cells per mouse). E) Overall survival of mice received surgeries and without surgeries with circulating tumor injection at the 5^th^ day after surgery (n = 11) (5 × 10^5^ cells per mouse). F) H&E staining for lung after Lewis lung carcinoma cells injection in mice received surgeries and without surgeries (5 × 10^5^ cells per mouse). G,H) Overall survival of mice received surgeries and without surgeries with circulating tumor cells (Lewis lung carcinoma cells/5 × 10^5^ cells per mouse, and melanoma B16‐F10 cells/1 × 10^5^ cells per mouse) injection at the 5^th^ day after surgery (n = 10). Graphs show mean ± SEM. *P < 0.05; **P < 0.01; ***P < 0.001. ns, not significant.

In order to identify whether the infiltration of inflammatory cells such as MDSCs would promote the osteosarcoma metastases in the appearance of circulating tumor cells, we designed a surgical model to simulate the impacts of these changes in the lung followed by tumor cell inoculation via tail vein. Tumor inoculation was performed at four time‐points: one day before surgery, one day after surgery, three days after surgery, or five days after surgery (**Figure** **4**A). Significantly promoted tumor growth was observed in the groups that received tumor cell inoculation after the surgery, corresponding to the extent of the post‐operative lung inflammation. Three weeks after cell inoculation, the lung weight and number of tumor nodules were assessed. The postoperative influx of circulating tumor cells led to significantly increased tumor metastasis in the lungs compared to the control group and the group inoculated before surgery (Figure 4B,C). This phenomenon was further confirmed by extending the sample size for mice that received circulating tumor cells inoculation five days after surgical trauma (Figure 4D). In order to identify the long‐term impacts of surgeries on osteosarcoma, we established a survival model, which indicates that after surgeries, the lung was susceptible to osteosarcoma circulating tumor cells and which contributed to a poor survival in mice (Figure 4E). However, the overall survival between groups challenging with osteosarcoma circulating tumor cells one day before surgery and non‐surgery mice have no significant differences (Figure S2A). To investigate whether enhanced tumor metastasis induced by surgical trauma is specific to osteosarcoma or applicable to other tumor types, we inoculated two different types of cancer cells (Lewis lung cancer cells and B16‐F10 melanoma cells) via tail vein after surgery. Similar results were observed with these solid tumor cells, which were associated with poorer survival in mice (Figure 4F–H).

In order to further explore the susceptibility of lungs after osteosarcoma surgeries under the presence of existing tumors, we developed an orthotopic osteosarcoma model on mice. After inoculation of osteosarcoma tumors in the proximal tibia, removal of primary tumors has prolonged survival than mice that did not receive surgical resection of the primary tumors (Figure S2B). However, most mice that received tumor resection died within six months after surgeries. An early study demonstrated an increase in postoperative metastasis with the duration between tumor inoculation and resection.^[^ ^19^ ^]^ In addition, the primary tumor would induce immunity to eliminate the disseminated tumor cells following the complete resection of primary tumors.^[^ ^21^ ^]^ To explore whether the lungs remain vulnerable to osteosarcoma cells after osteosarcoma surgeries in the presence of a primary tumor, we performed surgeries on the healthy leg three weeks after orthotopic osteosarcoma inoculation. Five days after surgery, traumatized mice were challenged with osteosarcoma cells via the tail vein, and the status of pulmonary metastases was analyzed when experimental mice became moribund (Figure S2D). The surgery group exhibited more pulmonary metastases than control groups (Figure S2E and S2F).

### 2.5 Peri‐operatively Anti‐inflammatory Intervention Inhibits the Formation of Pre‐metastatic Niche in Lung

**Figure 5:**
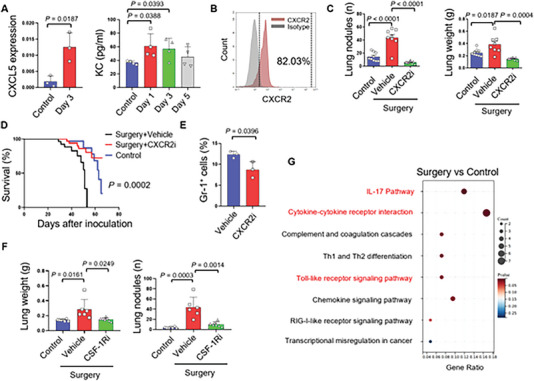
Perioperative immunomodulatory anti‐inflammation strengthens the resistance of lung to osteosarcoma circulating tumor cells. A) Real‐time PCR analysis of the level of CXCL5 in mice after surgeries (n = 3); and Luminex cytokine analysis of the KC after surgeries (n = 4). B) Flow cytometry analysis of the amount of Gr‐1^+^ MDSCs in lungs after surgery and the expression of CXCR2 on these cells. C) Statistics of the number and weight of pulmonary metastasis in mice received SB225002 (i.p 10 mg/kg) after surgery (n = 8) (5 × 10^5^ cells per mouse). D) Overall survival analysis of mice received different strategies of SB225002 administration during the perioperative period (n = 10) (5 × 10^5^ cells per mouse). E) Statistics of Gr‐1^+^ cells in lung of mice received SB225002 (i.p 10 mg/kg) and without treatment after surgery (n = 3). F) Statistics of the number and weight of pulmonary metastasis in mice received PLX3397 (p.o 30 mg/kg) after surgery (n = 6 to 7) (5 × 10^5^ cells per mouse). G) The KEGG pathway enrichment of the lungs at the 5^th^ day after surgery compared with the control group. Graphs show mean ± SEM. *P < 0.05; **P < 0.01; ***P < 0.001. ns, not significant.

In the subsequent experiments, to address the effect of suppressive MDSCs on forming pre‐metastatic niche, we investigate whether blockage of the inflammatory response or the infiltration of suppressive MDSCs would contribute to the resistance of circulating tumor cells in lung after surgery. We confirmed elevated levels of CXCL5 transcription and circulating KC secretion in mice (**Figure** **5**A), and IL‐8 in humans (Figure 1C), which are the chemokines and ligands of CXCR2. Besides, we observed that the persistent MDSCs in the lung during the perioperative period expressed CXCR2 (Figure 5B). The perioperative therapies of CXCR2 inhibitor avoid the acute phase after surgery tend to decrease metastasis after surgery (Figure 5C) and prolong the overall survival (Figure 5D). In addition, CXCR2 inhibition with SB225002 administration (i.p. 10 mg/kg) modulate the immune TME with decreased infiltration of Gr‐1^+^ myeloid cells (Figure 5E). We propose that immunomodulatory anti‐inflammatory approaches can enhance lung resistance to the circulating tumor cells after osteosarcoma surgeries. In addition, we observed the presence of inflammatory monocytes in the lungs at an early stage after surgery. CSF‐1R is a key target for monocytes and macrophages, and its inhibition has been shown to inhibit osteosarcoma metastasis.^[^ ^22^ ^]^ Indeed, perioperative administration of CSF‐1R inhibitor resulted in decreased tumor burden after surgery (Figure 5F). Additionally, we observed upregulation of Toll‐like receptor pathways in the lungs after surgeries, which are proposed to be activated by DAMPs (Figure 5G).

### 2.6 Mitochondrial DAMPs are Responsible for the Formation of Pre‐metastatic Niche after Surgery

**Figure 6:**
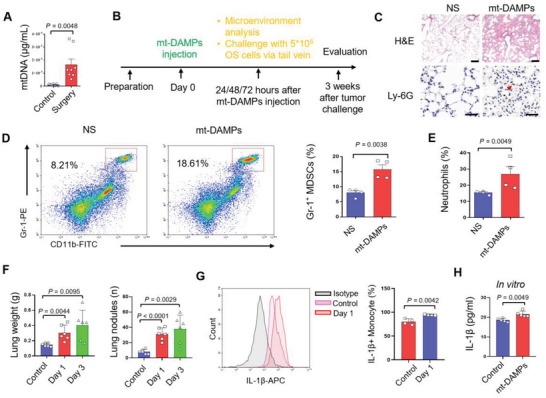
mt‐DAMPs initiated the postoperative inflammation and Gr‐1^+^ myeloid cells infiltration in lung. A) The quantitative analysis of the circulating mtDNA in mice subject to surgery (n = 8). B) The experimental design of mt‐DAMPs injection (mtDNA 5 µg) to mimic the postoperative mt‐DAMPs releasing. C) H&E staining and immunohistochemistry staining of Ly‐6G in lung from mice with mt‐DAMPs injection and without mt‐DAMPs injection (Scale bars, 50 µm for H&E, 100 µm for Ly‐6G IHC image). D) Flowcytometry reflecting the CD11b^+^Gr‐1^+^ MDSCs in mice with mt‐DAMPs injection and without mt‐DAMPs injection, and statistics of the MDSCs in the lung of mice with mt‐DAMPs injection and without mt‐DAMPs injection (n = 4). E) Statistics of the neutrophil in the lung of mice with mt‐DAMPs injection and without mt‐DAMPs injection (n = 4). F) Statistics of the weight and number of pulmonary metastases in mice who received mt‐DAMPs injection following circulating osteosarcoma cells injection one day and three days after mt‐DAMPs injection (n=6) (5 × 10^5^ cells per mouse). G) Flow cytometry analysis of the IL‐1β^+^ monocyte and statistics of the amount of IL‐1β^+^ monocyte in lungs after surgery(n = 4). H) ELISA quantify of the IL‐1β secretion after mt‐DAMPs stimulation on RAW264.7 monocytes (P<0.05). Graphs show mean ± SEM. *P < 0.05; **P < 0.01; ***P < 0.001. ns, not significant.

Trauma resulting from surgery elicits a systemic reaction characterized by an acute, non‐specific immune response.^[^ ^23^ ^]^ Patients with osteosarcoma undergoing amputation or limb‐salvage surgeries experience extensive tissue injuries, often accompanied by a brief period of mild fever following the procedures (Figure S3A and S3B). We hypothesized that the systemic response to trauma from these extensive surgical procedures in osteosarcoma patients may diminish the pulmonary resistance to circulating tumor cells. Moreover, inspired by the upregulation of the Toll‐like receptor pathway, we further speculated that DAMPs is an important factor driving this process.^[^ ^24^ ^]^ In a previous study, we found that circulating mitochondrial DAMPs (mt‐DAMPs) played a crucial role in triggering inflammatory responses following tissue injury.^[^ ^25^ ^]^Therefore, we designed primers to quantify the mitochondrial DNA (mtDNA) levels in a surgical mouse model and the results showed elevated levels of circulating mtDNA in mice that underwent surgeries (**Figure** **6**A).

In order to identify the influence of mt‐DAMPs on the inflammatory response in the lung, we simulate the DAMPs released after surgeries by direct injection of mt‐DAMPs via the tail vein (Figure 6B). Mice injected with mt‐DAMPs exhibited increased inflammatory infiltration in the lungs, as indicated by Ly‐6G‐positive cell populations (Figure 6C). Flow cytometry analysis revealed that mt‐DAMPs triggered the infiltration of MDSCs (Figure 6D) and neutrophils (Figure 6E) in the lungs. To further evaluate the impact of the mt‐DAMPs releasing on tumor metastasis, we challenged the mice with osteosarcoma cells one day and three days after mt‐DAMPs injection. The experimental group showed increased osteosarcoma metastases compared to the control group (Figure 6F). Additionally, the increase of monocyte in lung at the Day 1 after surgery presented as increased secretion of IL‐1β (Figure 6G). Then, we simulated the RAW264.7 monocyte with mt‐DAMPs, and the elevated IL‐1β was confirmed by ELISA assay (Figure 6H).

### 2.7 IL‐1β signal is Critical in the Settlement of Circulating Tumor Cells in Lungs after Surgery

**Figure 7:**
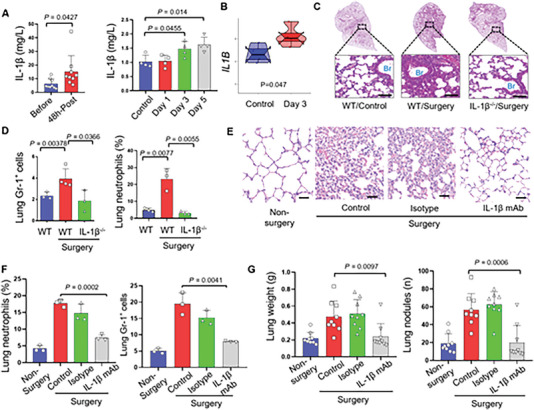
IL‐1β is a potential target attenuating postoperative pulmonary inflammation and Gr‐1^+^ myeloid cells infiltration. A) Quantitative analysis of the level of IL‐1β in patients before and after osteosarcoma surgeries, and in mice subject to surgery. Data are presented as means ± SEM (Student's *t*‐test; *n* = 4 – 9). B) the dumbbell plot of the transcriptional level of IL1b gene in lungs at the 3^rd^ after surgery and the control group (P<0.05). C) H&E staining of the lung from wild‐type mice without surgery, wild‐type mice received surgery, and IL‐1β^−/−^ mice received surgeries (Scale bars, 100 µm). D) Statistics of MDSCs and neutrophils in lung from wild‐type mice without surgery, wild‐type mice received surgery, and IL‐1β^−/−^ mice received surgeries (n = 3 to 4). E) H&E staining of the lung from mice subject to surgery, mice subject to surgery received isotype treatment, mice subject to surgery received IL‐1β mAb treatment (200 ug per mouse, i.p.) (Scale bars, 100 µm); and statistics of neutrophils in lungs. F) Statistics of neutrophile and MDSCs in lungs from mice subject to surgery, mice subject to surgery received isotype treatment, mice subject to surgery received IL‐1β mAb treatment (200 ug per mouse, i.p.). G) Statistics of the number and weight of pulmonary metastasis after K7M2 osteosarcoma cells challenging in mice received surgery, isotype and IL‐1β mAb treatment (n = 4 to 5) (5 × 10^5^ cells per mouse). Graphs show mean ± SEM. *P < 0.05; **P < 0.01; ***P < 0.001. ns, not significant.

IL‐1β is a well‐established and validated pathway that plays a critical role in inflammatory lung injury.^[^ ^26^ ^]^ Since we have detected the increased release of IL‐1β induced by circulating mt‐DAMPs (mtDNA and Formyl peptide) in the current study, the association between mt‐DAMPs, IL‐1β release and pre‐metastatic niche formation after osteosarcoma surgeries were further investigated. the levels of IL‐1β were elevated within three days after surgery in patients with osteosarcoma who underwent surgical treatment, as well as in traumatized mice (**Figure** **7**A). In addition, RNA‐seq of the lung tissues after surgery indicated the increased transcriptional level of the IL1B gene (Figure 7B).^[^ ^27^ ^]^

To evaluate the therapeutic potential of blocking the IL‐1β pathway in preventing the settlement of circulating tumor cells in the lungs after surgery, we established a surgical trauma model in IL‐1β genetic knocked‐out mice (IL‐1β^−/−^). H&E staining indicated that IL‐1β^−/−^ mice have attenuated inflammation in the lung at day five after surgeries (Figure 7C). In addition, the IL‐1β^−/−^ mice showed decreased levels of circulating neutrophils and MDSCs (Figure S4A), as well as reduced monocyte infiltration (Figure S4B). Moreover, the number of Gr‐1^+^ myeloid cells and neutrophils in the lungs decreased after surgeries (Figure 7D). Besides, the infiltration of CD3^+^CD8^+^ T cells increased in IL‐1β^−/−^ mice after surgeries (Figure S4C). In terms of anti‐inflammatory agent therapy, IL‐1β mAb also attenuated the lung inflammation, as reflected by H&E staining and flow cytometry analysis (Figure 7E). The inflammatory cytokines, including IL‐1β (Figure S5A), IL‐6 (Figure S5B), and the CXCR2 chemokine, KC (Figure S5C) decreased after IL‐1β mAb treatment. Similar to the IL‐1β^−/−^ mice after surgeries, the number of Gr‐1^+^ myeloid cells and neutrophil decreased in the lung after IL‐1β mAb treatment for wild‐type mice received surgeries (Figure 7F).

Having confirmed the ability of anti‐IL‐1β treatment in ameliorating the pre‐metastatic niche at the lung in mice subject to surgical trauma, we then investigated the therapeutic potential of anti‐IL‐1β during the perioperative period in traumatized mice. Subsequently, we challenged the IL‐1β^−/−^ mice with different solid tumor cells (melanoma B16‐F10 cells and Lewis lung carcinoma cells) via tail vein after surgeries. Two or three weeks later, these mice exhibited lower tumor burden of melanoma, and lung cancer (Figure S6A–D) than the corresponding wild‐type mice that underwent osteosarcoma surgeries. In addition, mice treated with IL‐1β mAb after surgery exhibited decreased metastasis (Figure 7G). Thus, this study has revealed the specific inflammatory mechanisms underlying the formation of pre‐metastatic niches in the lungs after local osteosarcoma surgeries, which are crucial for developing rational therapies aimed at improving the long‐term survival of osteosarcoma patients.

## 3 Discussion

Several observations concerned with osteosarcomas, surgical treatment, lung pre‐metastatic niches and mt‐DAMPs were made in the current study. The post‐operative metastasis in lung has been a challenge for osteosarcoma treatment after the curative primary tumor resection, however, the underlying mechanism for post‐operative lung metastatic relapse remains unclear. Our study revealed that, despite the immune‐regulatory effects of primary tumor, the surgical treatment itself could induce the distant pre‐metastatic niche in lung, which is characterized by the infiltration of MDSCs. Furthermore, the surgical treatment released mt‐DAMPs, that contributed to the pre‐metastatic niche formation and stimulated the upregulation of IL‐1β signal, which is critical in the settlement of circulating tumor cells in lungs after surgery. Our study revealed that the formation of pulmonary pre‐metastatic niche might be closely related to the surgical resection procedure in osteosarcoma treatment, which renders lung a favorable environment for circulating tumor cells to settle and is of significance for the development of related therapy.

A recent proof‐of‐concept study indicated that after surgery, more cancer cells were expelled into the circulation, especially after open radical surgeries for solid tumors.^[^ ^16b^ ^]^ In addition, the clearance of the circulating tumor cells during the perioperative period after surgical resection of the solid malignant tumor is associated with better prognosis.^[^ ^28^ ^]^ The current study provides direct evidence that osteosarcoma surgeries increase the opportunity for postoperative circulating tumor cell colonization in the lung, even in the presence of a primary tumor. Early studies demonstrated a slight increase in the number of pulmonary metastases following amputation of a normal extremity, whereas celiotomy and partial hepatectomy significantly increased lung metastases.^[^ ^29^ ^]^ This model supports our finding that surgical trauma could accelerate the settlement of circulating tumor cells. In this study, the tumor‐bearing leg was amputated two days after the trauma surgery to prevent further spread of circulating tumor cells into circulation. We proposed that these results can be attributed to the following reasons: the surgeries were performed relatively early after the inoculation of tumors when the tumor volume reached 8.0 mm^3^. Previous studies have shown that late surgery (at 22^nd^ day) after tumor incubation increased the risk of pulmonary metastasis compared to early surgery (at eighth day) due to the sufficient spread of circulating tumor cells into circulation following tumor growth.^[^ ^19^ ^]^ To investigate whether surgical trauma renders the lungs vulnerable to circulating tumor cells, we rechallenged the surgical mice with osteosarcoma cells. The amputation group showed an increased number of pulmonary metastases, supporting the idea that surgical trauma enhances the colonization of circulating tumor cells in the lungs, even in the presence of primary tumors.

An overwhelming inflammatory response occurs after surgical trauma, resulting in the release of internal danger matters, which the host immunity would recognizes and initiates a defense against.^[^ ^24^ ^]^ Among these matters, mitochondria have been extensively studied and are considered similar to bacteria.^[^ ^30^ ^]^ They are released following surgeries. Additionally, our hypothesis came from observing patients with severe trauma who exhibited elevated levels of circulating mtDNA, which correlated with clinical outcomes.^[^ ^31^ ^]^ We also considered the fact that circulating mt‐DAMPs contribute to inflammatory responses in injuries.^[^ ^32^ ^]^ Based on these observations, we proposed that mt‐DAMPs induce neutrophil influx in the lungs, thereby partially contributing to the formation of the pre‐metastatic niche. The conclusion of our study was supported by the evidence that some injuries would trigger pulmonary inflammation via releasing DAMPs.^[^ ^33^ ^]^ Besides, mt‐DAMPs causes lung injury with increased wet/dry ratio and endothelial permeability through neutrophil‐related pathways.^[^ ^32^, ^34^ ^]^ Thus, the host defense against these mt‐DAMPs causes inflammatory injuries in the lung,^[^ ^35^ ^]^ creating an environment in which circulating tumor cells released after surgeries can colonize. The critical role of MDSCs in pulmonary inflammation caused by mt‐DAMPs have been demonstrated in the current study. Previous research has shown that MDSCs are involved in systemic immune responses related to orthopedic conditions and that elevated MDSC levels after injury are correlated with poor functional healing outcomes.^[^ ^36^ ^]^ Once the DAMPs are released into circulation and reach the lungs, Gr‐1^+^ cells adhere to the endothelium, migrate into the interstitial and airspace, and perform phagocytic functions in the lung. This process is accompanied by a temporary lung injury characterized by an inflammatory response, edema, and increased permeability, representing a neutrophil‐derived lung injury under aseptic conditions.^[^ ^37^ ^]^

Anti‐inflammatory drugs have the ability to rapidly modify the tumor immune microenvironment, thereby enhancing immunogenicity.^[^ ^38^ ^]^ For example, in blunt trauma patients, the expression of IL‐1 in CXCR2^+^ lung neutrophils could be induced by IL‐5 expression by innate lymphocytes, culminating in early lung injury.^[^ ^39^ ^]^ Moreover, in patients with metastatic pediatric sarcomas the elevated levels of serum CXCR2 ligands and CXCL8 are associated with reduced survival rates. Disrupting CXCR2‐mediated trafficking of MDSCs improves the efficacy of anti‐PD1 treatment in pediatric sarcomas.^[^ ^40^ ^]^ Thus, CXCR2 blockade was proposed to be a possible strategy for remodeling the microenvironment after the circulating osteosarcoma cells colonization. For monocytes targeting, CSF‐1R inhibitor is a promising therapeutic option for advanced tumors and inflammatory disorders via anti‐inflammation and remodeling the immune‐response.^[^ ^41^ ^]^ For example, Chiauranib, a novel CSF‐1R kinase inhibitor associated with chronic inflammation, has shown favorable pharmacokinetic profiles and potential antitumor activity in patients with advanced solid tumors.^[^ ^42^ ^]^ In osteosarcoma, PLX3397 treatment concurrently depleted tumor‐associated macrophages and Foxp3^+^ regulatory T cells, that enhanced infiltration of CD8^+^ T cells into the microenvironments of both primary and metastatic osteosarcoma sites.^[^ ^43^ ^]^ In our study, preoperative administration of CSF‐1R inhibitor decreased the colonization of osteosarcoma circulating tumor cells in the lungs.

IL‐1β is a critical pro‐inflammatory cytokine involved in the innate immune response.^[^ ^44^ ^]^ Previously, celecoxib was proposed to improve the long‐term survival of patients with solid tumors after surgery. However, a long‐term clinical trial presented that the addition of celecoxib was proposed cannot the improve overall survival for patients who received tumor resection.^[^ ^45^ ^]^ We proposed that developing a direct target focusing on surgical damage‐induced inflammation could potentially enhance long‐term survival in osteosarcoma patients after surgeries.^[^ ^46^ ^]^ In our study, the lungs of IL‐1β^−/−^ mice displayed reduced inflammation and infiltration of Gr‐1^+^ myeloid cells after surgery, rendering them resistant to tumor cell colonization. Notably, IL‐1β has been demonstrated to mitigate the inflammation in septic lung injury^[^ ^26a^ ^]^ and ameliorate pulmonary ischemia‐reperfusion injury in clinical settings.^[^ ^47^ ^]^ Also, IL‐1β shows promise in preventing sepsis‐induced lung vascular injury in acute respiratory distress syndrome.^[^ ^48^ ^]^ Several monoclonal antibody products targeting IL‐1β have already entered clinical trials for the treatment of inflammatory diseases^[^ ^49^ ^]^ and cancer immunotherapy.^[^ ^50^ ^]^ Thus, targeting IL‐1β during the perioperative period emerges as a promising approach to improve long‐term survival after cancer surgery. Indeed, IL‐1β mAb show therapeutic potential in traumatized mouse models in our study.

Although previous study and the current study has suggested that trauma might be closely linked to tumor recurrence and distant metastasis, as well as inducing immunosuppression through the PD‐1/PD‐L1 pathway after surgery in patients.^[^ ^51^ ^]^ However, we must admit that surgical resection, indeed, would benefit the patient bearing osteosarcomas, especially for the early‐stage disease.^[^ ^52^ ^]^ Patients with high‐stage osteosarcoma, especially Enneking II disease, at which time, have a high risk of distant metastasis.^[^ ^52^, ^53^ ^]^ In our orthotopic model, most mice that received primary tumor resection died within three months after surgeries, although they achieved a significant longer‐term survival than tumor‐bearing mice without surgeries. So, the long‐term survival of these patients, in part, is determined by the treatment during the perioperative period. Therefore, in the current study, we have linked the systemic immune response to local surgical treatment and the influences on the immune microenvironment of distant organs and given the fact that we proved that injuries from osteosarcoma surgeries would facilitate the colonization of circulating tumor cells in the lung, we cannot rule out the impacts of surgical stress, as well as the primary tumor on this process. In addition, for clinical application, we cannot uniform the phenomenon that surgical resection of the primary tumors would cause the lung vulnerable to metastasis in every patient with osteosarcoma due to the high heterogeneity of this tumor. Moreover, the window for drug administration to improve the microenvironment in the lung needs further evaluation in clinical studies, as the responding speed and the homeostasis are different from that in mice after surgical trauma.

## 4 Conclusions 

The current study elucidated how the local surgical treatment of osteosarcomas, irrespective of the primary tumor, could contribute to the formation of pre‐metastatic niche in pulmonary microenvironment for circulating tumor cells settlement. The understanding of the underlying mechanism for postoperative lung metastasis in osteosarcomas is crucial for developing the related therapies during peri‐operative period and improving the long‐term survival of these patients.

## 5 Experimental Section

### Clinical Data Collection and Analysis

In order to evaluate the response to surgical treatment in patients with osteosarcoma, we collected the data of the perioperative period, including immune cells, cytokines, and inflammatory protein. The study protocol was approved by Ethics Committee of West China Hospital, Sichuan University (Chengdu, China). Written informed consent was obtained from participating patients (or their relatives) upon enrollment.

### Circulating Tumor Cells Detection and Analysis

Circulating tumor cells isolation and identification were performed according to the previous study.^[^ ^54^ ^]^ Here is the following strategy: for circulating tumor cells separation, 10 mL of peripheral blood was extracted, and the red blood cells in the peripheral blood were lysed first, then filtered these cells through a nano‐membrane. Based on the differences in size between tumor cells and white blood cells, circulating tumor cells separation and enrichment were performed; after the separation of circulating tumor cells, in order to type and identify the circulating tumor cells, a new method of multiple mRNAs in situ analysis (MRIA) was used for specific nucleic acid mapping of enriched circulating tumor cells to achieve the purpose of circulating tumor cells typing and identification. In this method, multiple RNA probes targeting circulating tumor cells were labeled at the same time. After the RNA probes were hybridized with the target gene, the detection sensitivity could be improved to single‐copy mRNA through the fluorescence signal amplification system.

### Cell Lines and Culture

Mice osteosarcoma cells K7M2 (Balb/c background), mice melanoma cells B16‐F10 (C57BL/6 background), and Lewis lung carcinoma cells (LL/2, C57BL/6 background) were purchased from the American Type Culture Collection (ATCC, Manassas, VA) and cultured with Dulbecco's modified Eagle's medium (DMEM) supplemented with amikacin and 10% fetal bovine serum in incubators at 37°C and 5% CO_2_.

### Animals

4‐ to 5‐week‐old female Balb/c, C57BL/6 mice were purchased from HFK Bioscience (China) and raised under specific pathogen‐free (SPF) conditions. All protocols were approved by the State Key Laboratory of Biotherapy Animal Care and Use Committee of Sichuan University in China. IL‐1β genetic knocked‐out (IL‐1β^−/−^) mice (C57BL/6 background) were obtained as a gift from The University of Tokyo, authenticated and raised under specific pathogen‐free conditions.

### Mimic the Clinical Surgery for Osteosarcoma in Mice

Amputation and limb‐salvage surgeries are the main types of surgical treatments for patients bearing osteosarcoma in the limb. Based on the previously reported trauma model, in which the mice were amputated after tumor incubation, we adapted the amputation surgery to mimic the clinical surgery for osteosarcoma in the mice model. The Surgical procedure is presented in Fig.s1.

### Flow Cytometry Analysis for Microenvironment

Fresh lung tissues and blood samples from experimental mice were managed into single‐cell suspensions and stained with CD45, CD11b, F4/80, CD206, Gr‐1, Ly6G, Ly6C, CD3, CD4, CD8, CD69, IL‐1β and CXCR2 (Table.s1). In detail, fresh lung tissues and blood samples from experimental mice were managed into single‐cell suspensions. The fresh lung tissues were cut into small pieces and then digested for 1 hour with 1% collagenase at 37°C. After centrifugation, single‐cell suspensions were obtained by grinding. Then they were treated with red cell lysate on ice for 10 minutes and washed twice with PBS. The blood samples were treated with red cell lysate on ice for 10 minutes and washed twice with PBS. All of the antibodies were purchased from BD Pharmingen™ (USA) or BioLegend, Inc. (USA). Flow cytometry analysis was performed on a FACS flow cytometer (ACEA NovoCyte^TM^, USA), and the data were analyzed using NovoExpress software.

### Cytokines Assay During Peri‐operative Period

Serum was collected from mice subject to surgical trauma at indicated points during peri‐operative period. Cytokines including IL‐1β, IL‐6, G‐CSF, KC, IL‐17A, IL‐10, and IL‐12p40 in 1:2 diluted serum samples from mice subject to surgical trauma were detected by Luminex assays according to the manufacturer's instructions.

### Immunohistochemical Analysis

Tissues were fixed in 4% paraformaldehyde for 48–72 hours and embedded in paraffin. The sections were baked for 1 h at 65°C, deparaffinized in xylene twice for 10 min, rehydrated in gradient ethanol for 2 min, and rinsed in deionized water twice for 5 min. Then, the sections were immersed with suitable citrate buffer for 3 min in an autoclave and incubated with goat serum for 20 min at 37°C after blocking with 3% H_2_O_2_ for 20 min at room temperature. The Ly‐6G primary antibody (1:400, Cat# GB11229, Servicebio, China) were added dropwise and incubated at 4°C overnight. Goat‐rabbit secondary antibody was added dropwise and incubated for 30 min at room temperature. The DAB developer was added dropwise and incubated for 3 min. Capture the digital images using cameras of the Case Viewer software.

### Wet to Dry Ratio of the Lung after Surgery

During surgeries‐induced lung injury in the perioperative period, lung edema was quantitated by determining wet: dry weight ratios as follows. A total of 5 lungs (Balb/c, 4‐ and 5‐weeks‐old, female) per group (Control, one day after surgery, three days after surgery, five days after surgery) were harvested after the surgical procedure and the designed observation time, then weighed. After the evaluation of wet weight, desiccated these lungs by incubation at 80°C overnight in a vacuum oven. They were then reweighed to determine the dry weight, and the wet: dry ratio was then calculated.

### Tail Vein Injection and Quantification of Tumor Nodules in Lungs

In vivo models were established by intravenous (i.v.) injection of K7M2 cells (approximately 5×10^5^ cells/0.1 mL serum‐free DMEM, Balb/c mice) or B16‐F10 cells (approximately 1×10^5^ cells/0.1 mL serum‐free DMEM, C57BL/6 mice or IL‐1β^−/−^ mice) or LL/2 cells (approximately 5×10^5^ cells/0.1 mL serum‐free DMEM, C57BL/6 mice or IL‐1β^−/−^ mice). The mice were sacrificed when they appeared moribund.

### Orthotopic Osteosarcoma Mice Models

Mice osteosarcoma cells, K7M2, were suspended using DMEM (Amikacin and fetal bovine serum‐free) at the concentration of *1* × *10^8^* cells/ml, in order to keep that each mouse was injected a number of *1* × *10^6^* cells at a volume of 10ul. For orthotopic injection, mice were anesthetized using isoflurane (2.5%) with an anesthesia machine (RWD, USA) and placed in a supine position. After operative area sterilization with 75% ethyl alcohol, a 28‐G½‐inch needle and a drilling motion were used for tumor suspension injection. The needle was inserted through the patellar ligament and into the anterior intercondylar area of the tibia. The needle was withdrawn, and a 1.0 ml syringe filled with cell suspension was used to inject 1 × 10^6^ cells into the previously drilled tibia tract slowly. Postoperatively, the mice were kept in a cage placed near a working electric oven until they recovered from anesthesia.

Overall survival analysis: In order to mimic the clinical situation, after the palpable of the orthotopic tumors (about three weeks after tumor injection), we performed tumor resection surgery as the experimental group, with mice did not receive tumor resection as the control group, to compare the overall survival of the two groups. If one mouse was dead during observation, the lung was obtained to analyze the reason for death.

Tumors cells rechallenging study: To analyze if surgical trauma would cause the lung vulnerable to osteosarcoma metastasis under the condition of primary tumor‐bearing, we performed the metastatic tumor cells rechallenging study. After three weeks of orthotopic tumor incubation, three groups were designed (Control group with no surgery performed and labeled as group 1; group 2 were mice that received amputation surgery on the affected leg; group 3 were mice that received amputation surgery on health leg), 5 days after surgery, 5*10^5^ osteosarcoma cells were injected via tail vein. Two weeks after tumor cells were rechallenging, and the mice were sacrificed to analyze the number of pulmonary metastases. After tumor cells were rechallenging, if mice dead, the lung was collected and calculated the number of pulmonary metastasis and record the time since tumor rechallenges.

### RNA‐seq Analysis and Bioinformatics for the Pulmonary Microenvironment after Surgery

Lungs from mice at different time points (Control, one day after surgery, three days after surgery, five days after surgery) after surgeries are collected and sored at liquid nitrogen prepared for RNA isolation and sequencing. RNA integrity was assessed using the RNA Nano 6000 Assay Kit of the Bioanalyzer 2100 system (Agilent Technologies, CA, USA). Total RNA was used as input material for the RNA sample preparations. Briefly, mRNA was purified from total RNA using poly‐T oligo‐attached magnetic beads. The library preparations were sequenced on an Illumina Novaseq platform, and 150 bp paired‐end reads were generated. Then, we used the cluster‐Profiler R package to test the statistical enrichment of differential expression genes in Gene Ontology (GO) pathways.

### Quantitative Real‐time PCR for Mitochondrial DNA

Peripheral blood from mice who received amputation surgery was collected one day after surgery to evaluate mtDNA levels. The mtDNA in the plasma was purified using the QIAamp DNA Blood Mini Kit (Qiagen) and quantified by qPCR performed with Taqman probes. The following primers were designed and synthesized by Invitrogen. The standard curve was created by analyzing serial dilutions of plasmid DNA inserted with the target PCR product (J01420, positions 2891‐3173).

*Mice primers and probe*:

**Table:** 

*Forward (5′‐3′)*	ACCTACCCTATCACTCACACTAGCA
*Reverse (5′‐3′)*	GAGGCTCATCCTGATCATAGAATG
*Probe (5′‐3′)*	FAM‐ ATGAGTTCCCCTACCAATACCACACCC ‐TAMRA

### mtDNA isolation and quantify

mtDNA was isolated from mouse liver and brain using a mtDNA isolation Kit (Abcam, Cambridge, MA, USA) following the standard procedure under sterile conditions. Mitochondria were isolated under sterile conditions. No protein contamination was found. The mtDNA was diluted in sterile water, quantified with NanoDrop 2000 UV‐Vis Spectrophotometer (Waltham, USA), and stored at −80°C. The endotoxin levels were below 0.25 EU/mL for all samples. The mtDNA was prepared for the following experiment.

### Mt‐DAMPs Simulation of Monocytes

Formyl peptide was purchased from Sigma‐Aldrich (F3506‐10MG). The mice RAW264.7 cells were placed into 6‐wells plates with 2*10^4^ cells each well. These cells were co‐incubated with mt‐DAMPs (5 µg mtDNA + *1* *µM* formyl peptide) for 8 hours. Then the cell supernatants were collected, and the IL‐1β ELISA kit (Absin, China) was used to analyze the level of IL‐1β following the manual instructions.

### mt‐DAMPs Injection Mimicking the DAMPs Releasing after Surgery

In order to mimic the mt‐DAMPs releasing after surgery and identify the role of these DAMPs in pulmonary inflammation after surgeries, we inject the mt‐DAMPs into circulation via the tail vein. Each mouse was injected with mt‐DAMPs containing a quantity of 5 µg mtDNA, which was suspended using saline solution. Mice received saline solution without mt‐DAMPs were designed as the control group. At one day, two days, and three days after mt‐DAMPs injection, mice were sacrificed for microenvironment evaluation, including neutrophils, MDSCs by flow cytometry. Also, the lungs from each group were fixed with 4.0% paraformaldehyde for H&E staining.

Also, in order to analyze the acceleration of mt‐DAMPs releasing on pulmonary metastasis of osteosarcoma, after one day, two days, and three days mt‐DAMPs injection, mice were injected with a 100 µl K7M2 cells suspension at a concentration of 5*10^6^ cells/ml via tail vein. Three to five weeks after tumor cell injection, mice were sacrificed for pulmonary metastasis evaluation.

### Real‐time Quantitative Polymerase Chain Reaction (PCR)

According to the procedures, total RNA was isolated utilizing the RNA simple Total RNA Kit (QIAGEN, China) and quantified with the NanoDrop 2000 UV‐Vis Spectrophotometer (Waltham, USA). Total RNA (1 µg per group) was reverse transcribed using a PrimeScript^TM^ RT Reagent Kit with gDNA Eraser (TaKaRa, Japan). GAPDH was used as an internal control in this research.

Primers were designed as follows:

CXCL5‐forward (*5*′‐GTTCCATCTCGCCATTCATGC‐*3*′);
CXCL5‐reverse (*5*′‐GCGGCTATGACTGAGGAAGG‐*3*′);
GAPDH‐forward (*5*′‐AACTTTGGCATTGTGGAAGG‐*3*′);
GAPDH‐reverse (*5*′‐ACACATTGGGGGTAGGAACA‐*3*′).

Quantitative real‐time PCR was performed using a Bio‐Rad CFX 96 with SSO Advanced^TM^ Universal SYBR® Green Supermix (Bio‐Rad, USA). All experiments were performed in triplicate.

### Therapeutic Experiments Study

The CXCR2 inhibitor, SB225002 (Selleck Chemicals, USA), and the CSF‐1R inhibitor, PLX3397 (WuXi AppTec, China), were used for the therapeutic study. Collectively, SB225002 and PLX3397 were dissolved according to the protocol from the manufacturer. SB225002 was administrated through i.p, 10 mg/kg during the perioperative period. PLX3397 was administrated through p.o, 30 mg/kg for 14 days after surgery. IL‐1β mAb (#BE0246, Bio X cell, USA) was administrated through i.p, *200* *µg* for each mouse, at the timepoint of 24 hours after surgery. 3–5 weeks after tumor injection, mice were sacrificed to evaluate the number and weight of pulmonary metastasis. Besides, the same‐designed groups were raised for overall survival analysis.

### Statistical Analysis

Statistics analyses were performed by using Prism 8.0 software (GraphPad, USA). Data are presented as means ± SEM. Comparisons of means between two groups were performed by unpaired, two‐tailed *t*‐test. Kaplan‐Meier survival analysis was used a log‐rank test to determine the differences. Normal distribution and similar variation of the data between experimental groups were examined for appropriateness before statistical tests were conducted. Results were considered statistically significant when *P* < 0.05.

## Conflict of Interest

The authors declare no conflict of interest.

## Author Contributions

F.T. and Y.T. contributed equally to this work. Y.Q.‐W., C.Q.‐T., and X.W.‐W. conceived the project and revised the manuscript. F.T. and Y.T. performed the experiments, analyzed the data and wrote the manuscript. F.T. conducted and established the animal models. T.X.‐L., W.Q.‐H., S.Y.‐C., H.H.‐S., L.M., and J.Y.‐Y performed experiments. L.Q.‐L., and H.Z. analyzed the RNA‐sequencing data. Y.Q.‐W., C.Q.‐T., and X.W.‐W. revised the manuscript. All authors helped improve the manuscript.

## Supporting information

Supporting Information

## Data Availability

The data that supports the findings of this study are available in the supplementary material of this article.
