# Supplementary material for: Surgical Treatment of Osteosarcoma Induced Distant Pre‐Metastatic Niche in Lung to Facilitate the Colonization of Circulating Tumor Cells
Source: Adv Sci (Weinh). 2023 Aug 16;10(28):2207518. doi: 10.1002/advs.202207518 (PMC10558698; doi:10.1002/advs.202207518)
Supplement: Supplementary file 1 — Supporting Information [file ADVS-10-2207518-s001.pdf]

ADVANCED SCIENCE
Open Access

Supporting Information

for *Adv. Sci.*, DOI 10.1002/advs.202207518

Surgical Treatment of Osteosarcoma Induced Distant Pre-Metastatic Niche in Lung to Facilitate the Colonization of Circulating Tumor Cells

Fan Tang, Yan Tie, Tian-Xia Lan, Jing-Yun Yang, Wei-Qi Hong, Si-Yuan Chen, Hou-Hui Shi, Long-Qing Li, Hao Zeng, Li Min, Yu-Quan Wei, Chong-Qi Tu and Xia-Wei Wei**

Surgical treatment of osteosarcoma induced distant pre-metastatic niche in lung to facilitate the colonization of circulating tumor cells

Fan Tang[1,2†], Yan Tie[1†], Tian-Xia Lan[1], Jing-Yun Yang[1], Wei-Qi Hong[1], Si-Yuan Chen[1], Hou-Hui Shi[1], Long-Qing Li[2], Hao Zeng[1], Li Min[2], Yu-Quan Wei[1], Chong-Qi Tu[2*], Xia-Wei Wei[1**]

[1]Laboratory of Aging Research and Cancer Drug Target, State Key Laboratory of Biotherapy, National Clinical Research Center for Geriatrics, West China Hospital, Sichuan University, No. 17, Block 3, Southern Renmin Road, Chengdu, 610041, Sichuan, People's Republic of China.
[2]Department of Orthopedics, Orthopedic Research Institute, West China Hospital, Sichuan University, No. 17, Block 3, Southern Renmin Road, Chengdu, 610041, Sichuan, People's Republic of China.

**Corresponding author. Chong-Qi Tu[*] and Xia-Wei Wei[**]

Email: [*]tucq@scu.edu.cn and [**]xiaweiwei@scu.edu.cn

†These authors contributed equally to this work.

Author Contributions: Y.Q.-W., C.Q.-T., and X.W.-W. conceived the project and revised the manuscript. F.T. and Y.T. performed the experiments, analyzed the data and wrote the manuscript. F.T. conducted and established the animal models. T.X.-L., W.Q.-H., S.Y.-C., H.H.-S., L.M., and J.Y.-Y performed experiments. L.Q.-L., and H.Z. analyzed the RNA-sequencing data. Y.Q.-W., C.Q.-T., and X.W.-W. revised the manuscript. All authors helped improve the manuscript.

Data availability statement: All data are available in the main text or supplementary materials.

Funding statement: This work was supported by the National Science Foundation for Excellent Young Scholars (32122052) and National Natural Science Foundation Regional Innovation and Development (No. U19A2003); the Special Program of China Postdoctoral Science Foundation 2020T130447; National Natural Science Foundation of China 82002847.

Conflict of interest disclosure: The authors declare no potential conflicts of interest.

Ethics approval statement: All animal experiments were performed as well as the clinical samples were collected in accordance with protocols approved by the West China Hospital, Sichuan University Research Ethics Committee.

Patient consent statement: Written informed consent was obtained from participating patients (or their relatives) upon enrollment.

Figure S1 Pulmonary microenvironment after osteosarcoma surgery.

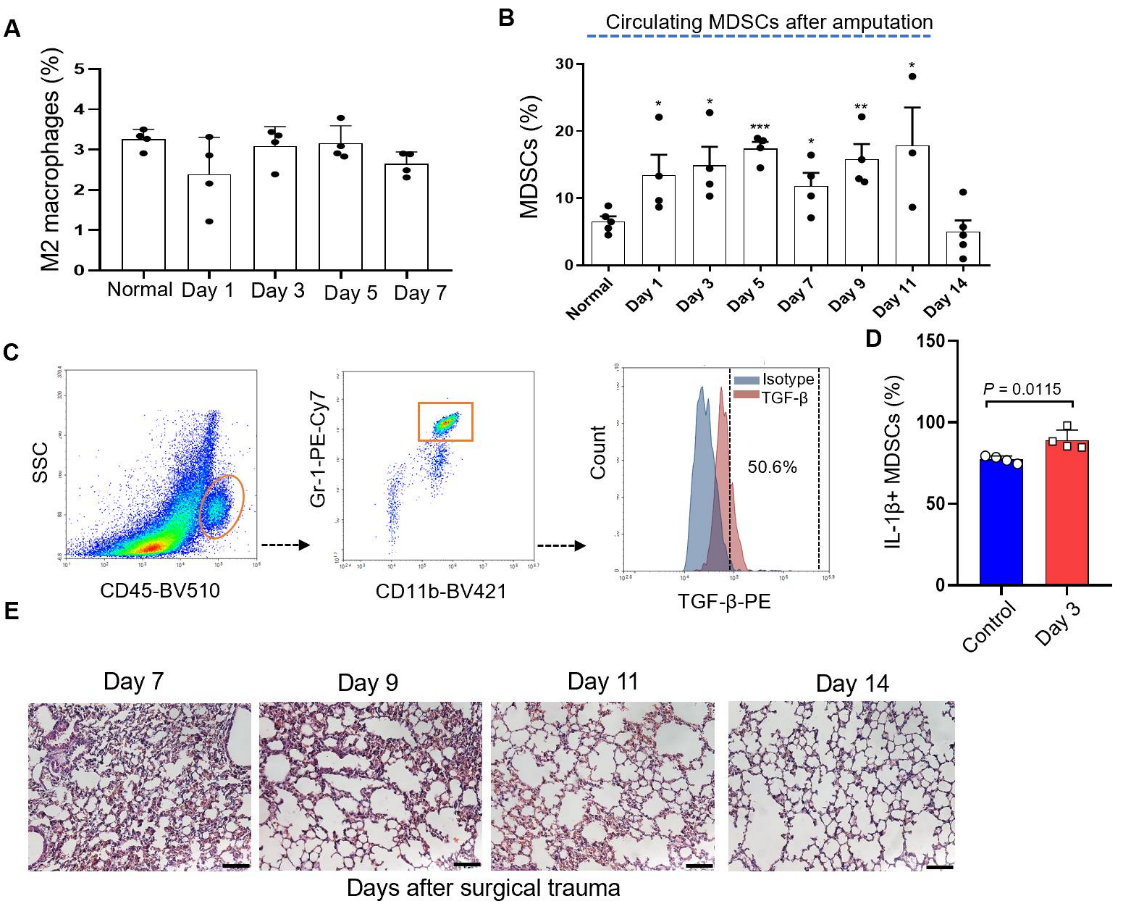

(A) Statistics of M2-macrophages in the lungs 1 day, 3 days, 5 days, and 7 days after amputation surgery. **(B)** Statistics of circulating MDSCs before and after amputation surgery. **(C)** The gating for MDSCs and the secretion of TGF-β in the lung by flow cytometry. **(D)** Flow cytometry analysis of the IL-1β^+ MDSCs and statistics of the amount of IL-1β^+ monocyte in lungs after surgery(n=4). **(E)** H&E staining of the lungs in mice 7

days, 9 days, 11 days, 14 days after surgical trauma (Scale bars, 50μm). Graphs show mean ± SEM (n = 4). *$P < 0.05$; **$P < 0.01$; ***$P < 0.001$. ns, not significant.

Figure S2 Lungs are vulnerable to circulating tumor cells in surgical traumatized mice.

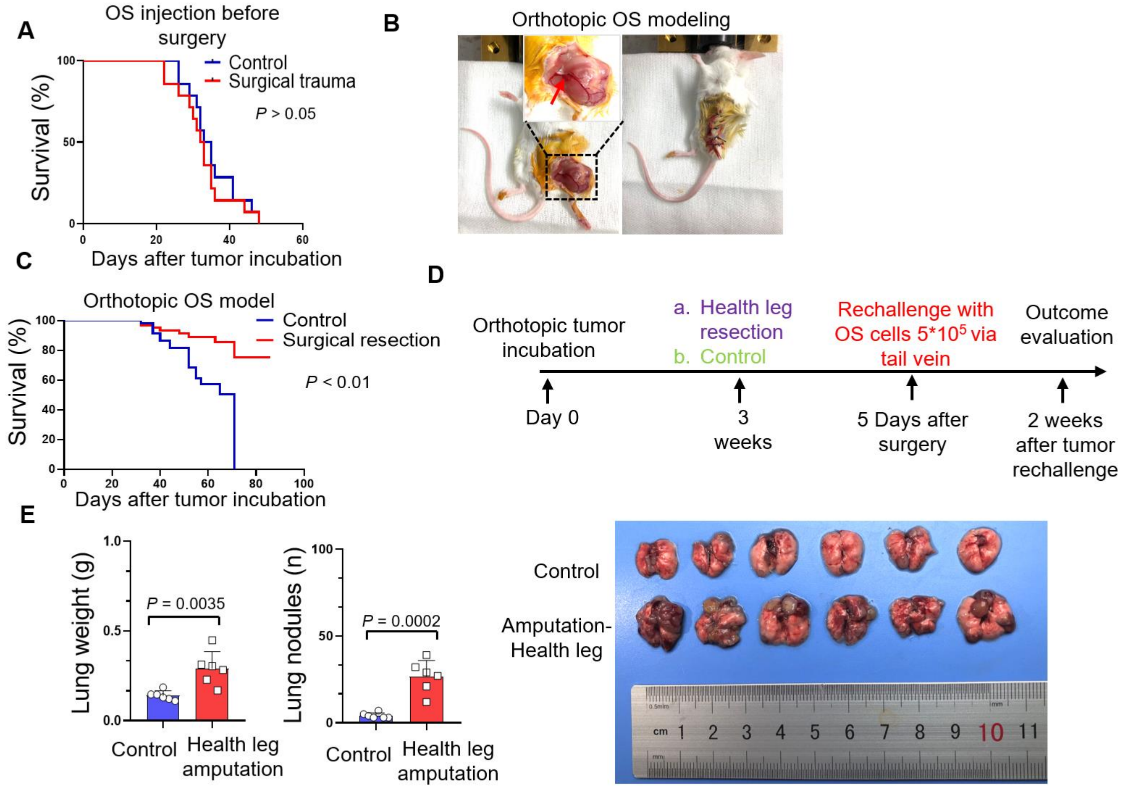

(A) Overall survival between mice without surgery and mice subject to surgery with circulating tumor cells injection three days before the surgery (n=14). **(B)** Images of amputation surgery for orthotopic tumor mice model. The arrow indicated the supplying vessels for the hind limb, which would be ligature before removal of the limb or the tumor-bearing limb. **(C)** The overall survival in orthotopic mice model between groups received tumor resection and without surgical resection (n=10 to 18). **(D)** The timeline of circulating tumor rechallenging experiment on orthotopic mice model. **(E)** Statistics of the number and weight of pulmonary metastasis, as well as the images of tumor-bearing lungs for circulating tumor cells rechallenging experiment on the orthotopic model (n=6). Graphs show mean ± SEM. **P* < 0.05; ***P* < 0.01; ****P* < 0.001. ns, not significant.

Figure S3. Osteosarcoma patients after surgical treatment were with increased temperature.

A

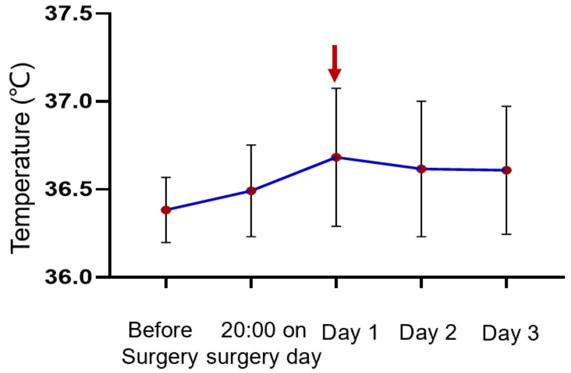

B

Cases	Gender	Age (Years)	Tumor location	Surgical treatment
1	Male	50	Proximal tibia	Limb salvage
2	Male	12	Distal femur	Limb salvage
3	Male	15	Distal femur	Limb salvage
4	Male	14	Distal femur	Limb salvage
5	Female	27	Distal femur	Amputation
6	Male	19	Distal femur	Limb salvage
7	Male	16	Distal femur	Amputation
8	Female	47	Distal tibia	Amputation
9	Female	4	Distal femur	Amputation
10	Female	16	Distal femur	Limb salvage
11	Male	13	Distal femur	Amputation
12	Female	14	Distal femur	Limb salvage

(A) The tendency of the temperature in patients with osteosarcoma after surgical treatment (n=12). **(B)** The basic information of the included patients for temperature observation during the perioperative period.

Figure S4 Blocking IL-1β signal remodeled the pulmonary microenvironment.

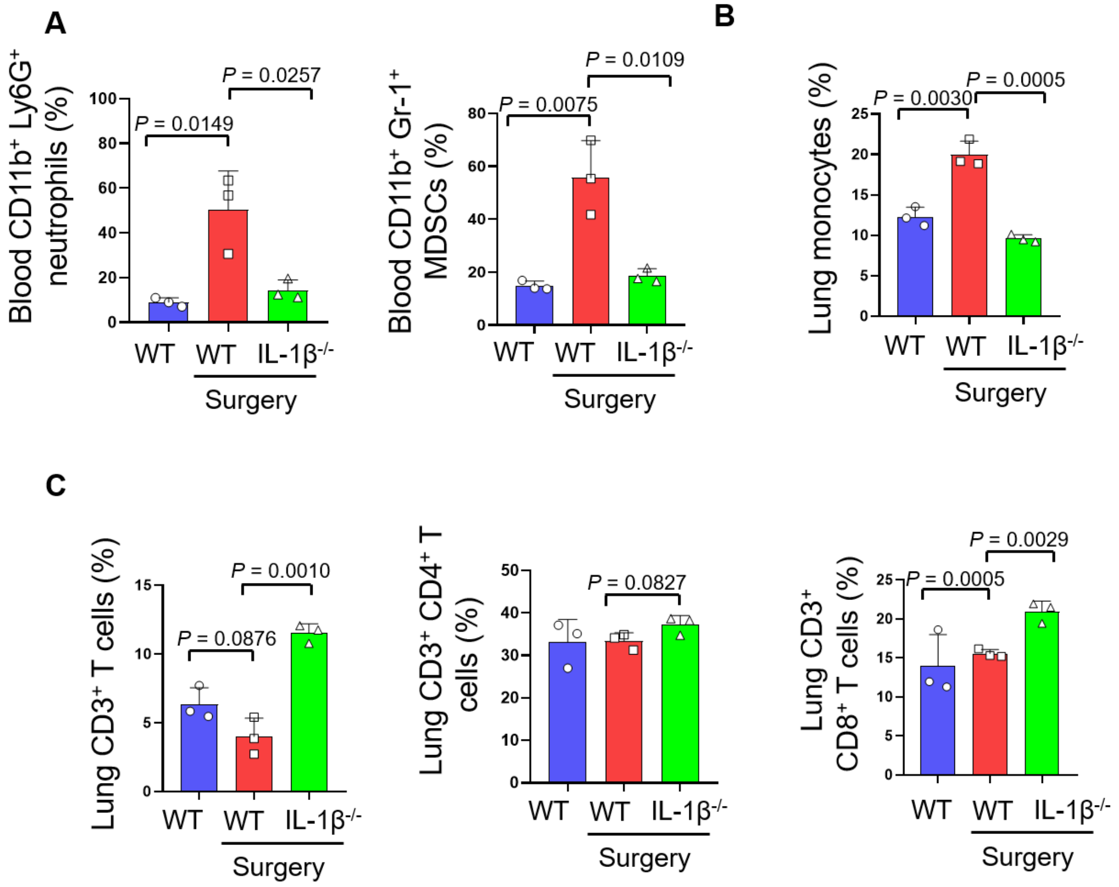

(A) Statics of the circulating neutrophil and MDSCs in wild-type mice without surgery, wild-type mice received surgery, and IL-1$\beta^{-/-}$ mice received surgeries. **(B)** Statics of the pulmonary monocytes in wild-type mice without surgery, wild-type mice received surgery, and IL-1$\beta^{-/-}$ mice received surgeries. **(C)** Statistics of $CD3^{+}$ T cells, $CD3^{+}CD4^{+}$ T cells, $CD3^{+}CD8^{+}$ T cells in lung from wild-type mice without surgery, wild-type mice received surgery, and IL-1$\beta^{-/-}$ mice received surgeries. Graphs show mean ± SEM (n = 3). *P < 0.05; **P < 0.01; ***P < 0.001. ns, not significant.

Figure S5. Pro-inflammatory cytokines decreased after IL-1β mAb treatment on surgically traumatic mice

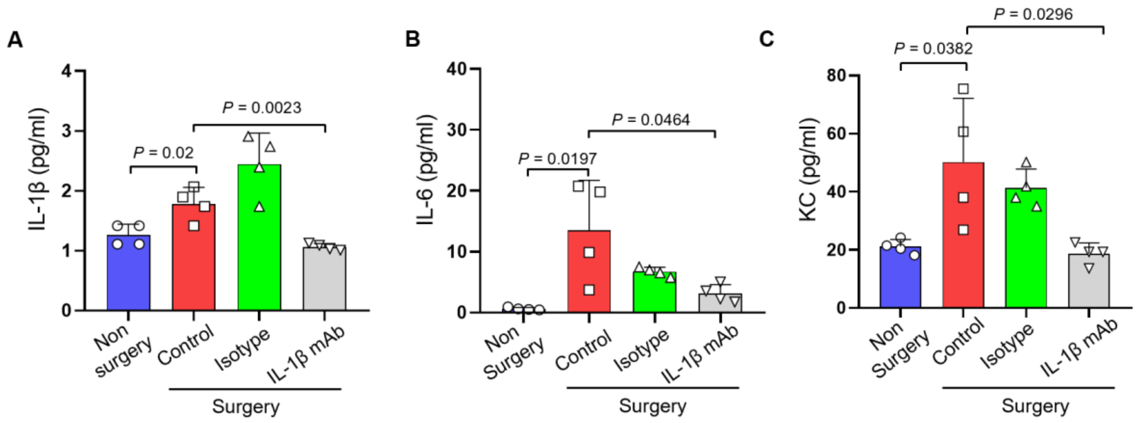

(A and **B)** Statistics of the concentration of IL-1β and IL-6 on surgical mice received IL-1β mAb treatment. **(C)** Statistics of the concentration of KC on surgical mice received IL-1β mAb treatment. Graphs show mean ± SEM (n = 4). *P < 0.05; **P < 0.01; ***P < 0.001. ns, not significant.

Figure S6. IL-1β$^{-/-}$ mice are resistant to circulating tumor cells after surgery.

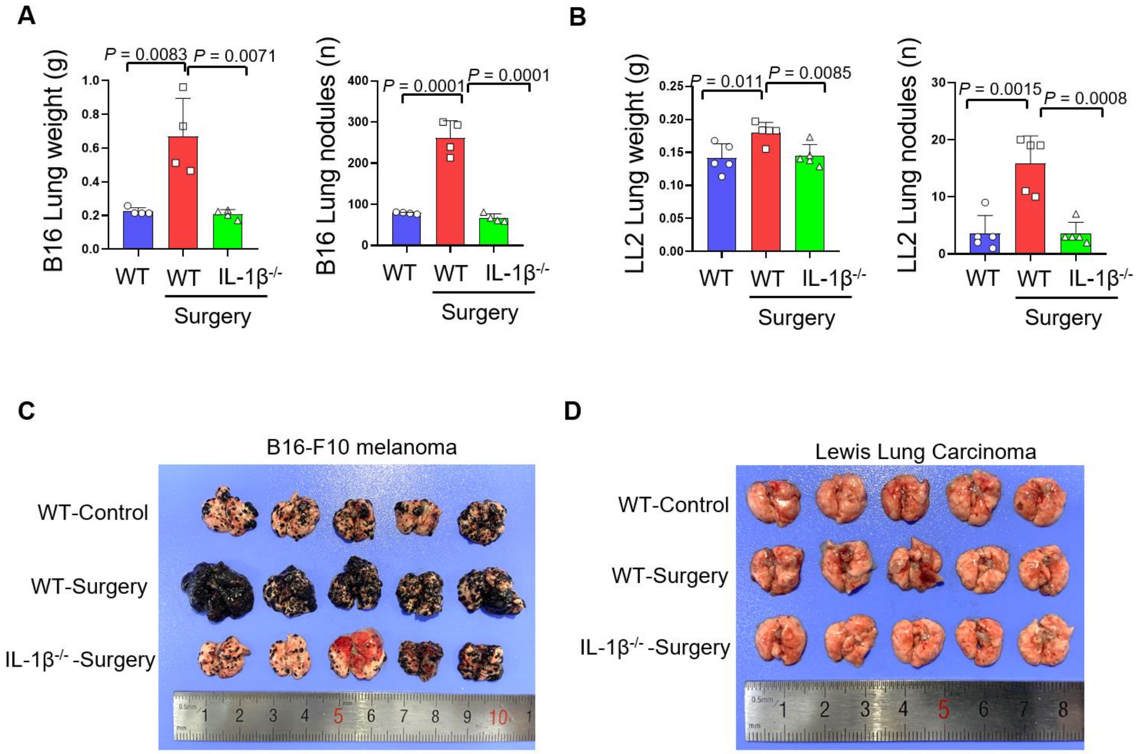

(A and **B)** Statistics of the number and weight of pulmonary metastasis after B16-F10 melanoma and Lewis lung carcinoma cells challenging in wild-type mice without surgery, wild-type mice received surgery, and IL-1β$^{-/-}$ mice received surgeries. **(C** and **D)** Images of the tumor-bearing lungs of B16-F10 melanoma and Lewis lung carcinoma in wild-type mice without surgery, wild-type mice received surgery, and IL-1β$^{-/-}$ mice received surgeries. Graphs show mean ± SEM (n = 4–5). *P < 0.05; **P < 0.01; ***P < 0.001. ns, not significant.

Table S1. Components of flow cytometry antibodies.

MDSCs

Marker	Fluorophore	Dilution	Source
CD45	BV510	1:100	BD Biosciences
CD11b	FITC	1:100	BioLegend
Gr-1	PE/Cy7	1:100	BioLegend
CXCR2	BV421	1:50	BD Biosciences

Neutrophils and Monocytes

Marker	Fluorophore	Dilution	Source
CD45	FITC	1:100	BioLegend
CD11b	BV421	1:100	BD Biosciences
Ly6G	PE	1:100	BioLegend
Ly6C	PerCP/Cy5.5	1:100	BioLegend
IL-1β	APC	1:100	eBioscience

Macrophages

Marker	Fluorophore	Dilution	Source
CD45	FITC	1:100	BioLegend
CD11b	BV421	1:100	BD Biosciences
F4/80	APC	1:100	BioLegend
CD206	PE	1:100	BioLegend
CD11c	PerCP/Cy5.5	1:100	BioLegend

T cells

Marker	Fluorophore	Dilution	Source
CD3	PE	1:100	BioLegend

CD4	APC	1:100	BioLegend
CD8	BV421	1:100	BioLegend
CD69	PerCP/Cy5.5	1:100	BioLegend
